# Association of subcortical gray-matter volumes with life-course-persistent antisocial behavior in a population-representative longitudinal birth cohort

**DOI:** 10.1017/S0954579421000377

**Published:** 2021-10-18

**Authors:** Christina O. Carlisi, Terrie E. Moffitt, Annchen R. Knodt, HonaLee Harrington, Stephanie Langevin, David Ireland, Tracy R. Melzer, Richie Poulton, Sandhya Ramrakha, Avshalom Caspi, Ahmad R. Hariri, Essi Viding

**Affiliations:** 1 Division of Psychology and Language Sciences, University College London, London, UK; 2 Department of Psychology and Neuroscience, Duke University, Durham, NC, USA; 3 Social, Genetic and Developmental Psychiatry Centre, Institute of Psychiatry, Psychology & Neuroscience, King’s College London, London, UK; 4 PROMENTA, Department of Psychology, University of Oslo, Oslo, Norway; 5 School of Criminology, University of Montreal, Quebec, Canada; 6 Dunedin Multidisciplinary Health and Development Research Unit, Department of Psychology, University of Otago, Dunedin, New Zealand; 7 New Zealand Brain Research Institute, Christchurch, New Zealand; 8 Department of Medicine, University of Otago, Christchurch, New Zealand; 9 Brain Research New Zealand – Rangahau Roro Aotearo Centre of Research Excellence, Dunedin, New Zealand

**Keywords:** antisocial behavior, conduct disorder, development, longitudinal, structural MRI

## Abstract

Neuropsychological evidence supports the developmental taxonomy theory of antisocial behavior, suggesting that abnormal brain development distinguishes life-course-persistent from adolescence-limited antisocial behavior. Recent neuroimaging work confirmed that prospectively-measured life-course-persistent antisocial behavior is associated with differences in cortical brain structure. Whether this extends to subcortical brain structures remains uninvestigated. This study compared subcortical gray-matter volumes between 672 members of the Dunedin Study previously defined as exhibiting life-course-persistent, adolescence-limited or low-level antisocial behavior based on repeated assessments at ages 7–26 years. Gray-matter volumes of 10 subcortical structures were compared across groups. The life-course-persistent group had lower volumes of amygdala, brain stem, cerebellum, hippocampus, pallidum, thalamus, and ventral diencephalon compared to the low-antisocial group. Differences between life-course-persistent and adolescence-limited individuals were comparable in effect size to differences between life-course-persistent and low-antisocial individuals, but were not statistically significant due to less statistical power. Gray-matter volumes in adolescence-limited individuals were near the norm in this population-representative cohort and similar to volumes in low-antisocial individuals. Although this study could not establish causal links between brain volume and anti-social behavior, it constitutes new biological evidence that all people with antisocial behavior are not the same, supporting a need for greater developmental and diagnostic precision in clinical, forensic, and policy-based interventions.

## Introduction

Young people who engage in persistent, pervasive antisocial behavior are often diagnosed with conduct disorder and are more likely to be incarcerated and have poor physical and mental health as adults (Rivenbark et al., 2018). Longitudinal cohort studies have demonstrated marked individual differences in the age of onset of antisocial behavior and its stability over time. A relatively small proportion of children (around 10%) display early-onset “life-course-persistent” antisocial behavior that continues into adulthood whereas, for a larger proportion (over 25%), antisocial behavior arises in adolescence but is limited to this pre-adult period (“adolescence-limited” antisocial behavior) (Moffitt, 2018). The Developmental Taxonomy Theory (Moffitt, 1993) distinguishes these patterns of antisocial behavior. The theory has been influential in guiding early-years prevention, juvenile justice policies, as well as clinical research and practice, such that the fourth edition of the *Diagnostic and Statistical Manual of Mental Disorders* (DSM) first incorporated the distinction between childhood-onset versus adolescent-onset conduct disorder (APA, 2000).

Longitudinal studies have found evidence for early behavioral and neuropsychological impairments in individuals on the life-course-persistent trajectory, including difficult temperament, low verbal IQ, poor executive function, and poor memory (Moffitt & Caspi, 2001). This suggests that those on the life-course-persistent trajectory have neuropsychological vulnerabilities that – alongside environmental risk factors – compound their difficulty in gaining prosocial skills to promote desistance from antisocial behavior. Consistent with these behavioral and neuropsychological vulnerabilities, developmental taxonomy theory specifies abnormal brain development as a key feature of those with life-course-persistent but not adolescence-limited antisocial behavior (Moffitt, 1993), suggesting that neuroimaging investigations of brain structure would be a useful target for understanding the mechanisms defining these groups. Recently, we examined differences in broad cortical anatomy in a large cohort of prospectively-defined antisocial individuals and found evidence of localized thinner cortex and broadly smaller surface area in individuals with life-course-persistent but not adolescence-limited antisocial behavior (Carlisi et al., 2020). While the findings of widespread differences in cortical anatomy are notable and add to the broader picture of life-course-persistent antisocial behavior, subcortical structures have perhaps been more strongly implicated in the pathology of antisocial behavior. For example, cross-sectional neuroimaging and neuropsychological studies in antisocial children – particularly those with callous-unemotional traits who tend to grow up to be among the most severe antisocial adults – have implicated abnormalities in behaviors including low emotional responsiveness to rewards and to others’ distress and poor reinforcement learning (Viding & McCrory, 2012), alongside differences in the subcortical regions underpinning them, including the amygdala, thalamus, and caudate (Fairchild et al., 2009, 2011, 2013; Passamonti et al., 2010; Rogers & De Brito, 2016). These subcortical regions are connected with cortical regions (including orbitofrontal, ventromedial, and dorsolateral prefrontal cortex) and the anterior cingulate cortex supporting more complex processes including decision making, impulsivity, emotional responses, and aggression, which are also implicated in the neurocognitive profile of persistent antisocial behavior (Blair, 2016; Goldin, McRae, Ramel, & Gross, 2008; Lamm, Decety, & Singer, 2011; Shigemune et al., 2010). However, it is interesting to note that the direction of effects, particularly in subcortical regions, seems to differ in child versus adult studies, and existing consensus in the literature on abnormalities in subcortical regions is lacking. Therefore, we now extend our prior research to investigate subcortical structures in this cohort with prospectively-tracked antisocial behavior.

To our knowledge, only one study has compared subcortical brain structures as a function of differences in the age of onset of antisocial behavior; that study reported lower amygdala volume in antisocial individuals, regardless of the age of onset (Fairchild et al., 2011). However, antisocial behavior was not assessed prospectively across the life course in that study (groups were based on retrospective reports from one occasion) and the sample was relatively small and predominantly middle class. A more comprehensive analysis is thus needed to evaluate antisocial individuals whose behavior has been ascertained using developmental information. Moreover, it is important to evaluate whether cross-sectional retrospective studies of antisocial behavior may have overlooked brain-based differences between developmental trajectories of antisocial behavior.

We analyzed structural magnetic resonance imaging (MRI) data collected at age 45 years from 672 members of the Dunedin Study (Poulton, Moffitt, & Silva, 2015) who were previously subtyped using eight repeated assessments of behavior from ages 7–26 years as exhibiting one of three developmental trajectories: (a) life-course-persistent antisocial behavior, (b) adolescence-limited antisocial behavior or (c) low levels of antisocial behavior (henceforth referred to as “low-antisocial”). Building on our initial study of cortical anatomy in these Dunedin Study members (Carlisi et al., 2020), we focused on gray-matter volume of subcortical structures. We hypothesized that, relative to the low-antisocial group, the life-course-persistent – but not adolescence-limited group – would show nonspecific patterns of absolute lower gray-matter volume across multiple subcortical regions, particularly the amygdala, dorsal striatum (i.e., caudate, putamen), ventral striatum (i.e., accumbens), and brain stem, which, in smaller studies, have been implicated in the neuropsychological deficits of antisocial behavior (Blair, 2016; Fairchild et al., 2011; Lundwall et al., 2017).

## Method

### Participants

The participants of this study are members of the Dunedin Study, a longitudinal investigation of a population-representative birth cohort. Participants (*N* = 1,037; 91% of eligible births; 52% male) were all infants born between April 1972 and March 1973 in Dunedin, New Zealand, who participated in the first assessment at age 3 years (Poulton et al., 2015). The cohort represented the full range of socioeconomic status in New Zealand’s South Island and, as adults, matched the New Zealand national health and nutrition survey on key adult health indicators and the New Zealand census of citizens of equivalent age on educational attainment. The cohort is primarily white (93%) (Poulton et al., 2015). Assessments were carried out at birth and at ages 3, 5, 7, 9, 11, 13, 15, 18, 21, 26, 32, 38, and, most recently, 45 years (completed April 2019). The relevant ethics committees approved each phase and written informed consent was obtained from all participants. Of the 1,037 original Dunedin Study members, 997 were alive and 938 (94%) took part at age 45 years. Of these, 875 (441; 50.4% male) underwent scanning. The scanned study members did not differ from other living study members on childhood socioeconomic status or childhood IQ (see Supplementary Material (Appendix 1)).

### Antisocial behavior

The Study members were previously classified as following a trajectory of life-course-persistent antisocial behavior (9%), adolescence-limited antisocial behavior (18%) or low-antisocial behavior (50%), as detailed by Odgers et al. (2008). Growth mixture modeling was applied, stratified by sex, to six facets of antisocial behavior assessed in all study members at 7, 9, 11, 13, 15, 18, 21, and 26 years of age: physical fighting, bullying, destroying property, lying, stealing, and truancy (or chronic work absenteeism). Based on repeated clinical assessments at these intervals, the life-course-persistent group met conduct disorder diagnostic criteria at 7, 9, 11, 13, and 15 years, while the adolescence-limited group only met criteria at age 15 years (Odgers et al., 2008). After age 26 years, the two antisocial groups continued to diverge in their patterns of antisocial behavior. According to nationwide records, between 26–38 years of age, the life-course-persistent individuals were convicted an average of 5.3 times, the adolescence-limited group 1.1 times, and the low-antisocial group 0.31 times (Moffitt, 2018).

### MRI data acquisition and processing

Structural MRI images were processed with FreeSurfer version 6.0 and mean gray-matter volumes of 10 bilateral subcortical structures (accumbens, amygdala, brain stem, caudate, cerebellum, hippocampus, pallidum, putamen, thalamus, and ventral diencephalon) were extracted using the automatic segmentation (‘aseg’) step. Segmentation accuracy was confirmed by visual inspection of the aseg labels overlaid on the volumes.

Each participant was scanned using a MAGNETOM Skyra (Siemens Healthcare GmbH) 3T scanner equipped with a 64-channel head/neck coil at the Pacific Radiology imaging center in Dunedin, New Zealand. High-resolution *T*_1_-weighted images were obtained using an MP-RAGE sequence with the following parameters: repetition time (TR) = 2,400 ms; echo time (TE) = 1.98 ms; 208 sagittal slices; flip angle = 9°; field of view (FOV) = 224 mm; matrix = 256 × 256; slice thickness = 0.9 mm with no gap (voxel size = 0.9 × 0.875 × 0.875 mm); total scan time = 6 min and 52 s. Three-dimensional fluid-attenuated inversion recovery (FLAIR) images were obtained with the following parameters: TR = 8,000 ms; TE = 399 ms; 160 sagittal slices; FOV = 240 mm; matrix = 232 × 256; slice thickness = 1.2 mm (voxel size = 0.9 × 0.9 × 1.2 mm); total scan time = 5 min and 38 s. In addition, a gradient echo field map was acquired with the following parameters: TR = 712 ms; TE = 4.92 and 7.38 ms; 72 axial slices; FOV = 200 mm; matrix = 100 × 100; slice thickness = 2.0 mm (voxel size = 2 mm isotropic); total scan time = 2 min and 25 s.

*T*_1_-weighted and FLAIR images were corrected for readout distortion using the gradient echo field map, co-registered, brain-extracted, and aligned together in the native *T*_1_ space using boundary-based registration (Greve & Fischl, 2009). The FLAIR images were included to improve the pial surface for cortical segmentation in the FreeSurfer recon-all pipeline. Images were then processed with a custom FreeSurfer recon-all pipeline that was optimized for structural MRI with resolution higher than 1 mm isotropic. Finally, the recon-all outputs were converted into CIFTI format and registered to a common 32k_FS_LR mesh using MSM-sulc (Robinson et al., 2014). MRI processing was done blind to antisocial trajectory group membership.

Subcortical volumes were calculated based on FreeSurfer’s aseg program. A study conducted and cross-validated by five tracing experts comparing FreeSurfer’s aseg results of subcortical structure labeling and volume calculation with the results of manual tracing showed that agreement between the automated and manual labeling was comparable to the agreement (inter-rater reliability) obtained by comparing the manual labeling across the five experts. Moreover, there was no discernible bias in the automated gray-matter volume measurements, which were statistically indistinguishable from the manually computed volumes (Fischl et al., 2002). Supplementary Figure 1 shows the automatic segmentation results from FreeSurfer’s aseg program overlaid on a single study member’s *T*_1_-weighted structural scan.

### Childhood conditions and adult experiences co-occurring with antisocial behavior

In secondary analyses, we tested if the following co-occurring experiences and conditions accounted for differences in subcortical gray-matter volume between groups: childhood socioeconomic deprivation; adverse childhood experiences; impaired neuropsycho-logical functioning (indexed by IQ); history of head injury; serious mental illness (schizophrenia); drug/alcohol dependence.

### Childhood socioeconomic status

Childhood family socioeconomic status was defined as the mean of the highest occupational status level of either parent across study assessments from the participant’s birth through to age 15 years, measured using the Elley-Irving Scale (Elley & Irving, 1976), which assigns occupations into one of six socioeconomic status groups (ranging from 6 = *professional* to 1 = *unskilled laborer*; *M* = 3.75, *SD* = 1.14).

### Adverse childhood experiences

Adverse childhood experiences were assessed through 10 categories of childhood adversity introduced by the CDC-Kaiser Permanente adverse childhood experiences study (Felitti et al., 1998) – five types of child harm (physical abuse, emotional abuse, physical neglect, emotional neglect, and sexual abuse) and five types of household dysfunction (incarceration of a family member, household substance abuse, household mental illness, loss of a parent, and household partner violence). Prospectively-ascertained adverse childhood experiences were determined for each study member from records collected at ages 3, 5, 7, 9, 11, 13, and 15 years (Reuben et al., 2016). These records included social services visits, structured interviews with the Study member and their parents, observed interactions between study members and parents, self-reports collected from parents regarding parental criminality, notes from home visits, and notes from teachers asked about the wellbeing of study members. Prospectively-ascertained adverse childhood experiences were available for all 861 participants with usable neuroimaging data. Data were coded 0, 1, 2, 3, or 4+ for all analyses in the current study.

### Childhood cognitive functioning

Childhood cognitive functioning was assessed via IQ scores derived in late childhood (at ages 7, 9, and 11 years), when the Study members were administered the Wechsler Intelligence Scale for Children – Revised (WISC-R) (Wechsler, 1974). Scores from the three WISC-R administrations were averaged to yield a single, reliable measure of cognitive function.

History of head injury (self-reported head injury requiring hospital treatment) and DSM diagnosis of serious mental illness (schizophrenia) and drug/alcohol dependence were assessed at age 45 years during clinical interviews.

### Statistical analyses

Statistical analyses were conducted in R version 3.5.0. Separate ordinary least-squares regression models were used to make pair-wise FDR-corrected comparisons (FDR = false discovery rate) between each group (Benjamini & Hochberg, 1995). Bilateral volume was calculated as an average between volumes for left and right hemispheres. Sex was included as a covariate in all the analyses. Secondary analyses including total brain volume as a covariate examined regional volume differences relative to differences in overall brain size. As a result of lower total brain volumes in antisocial individuals, differences in absolute regional subcortical volumes are likely to be attenuated when total brain volume is controlled for.

This study/analysis plan was pre-registered (https://sites.google.com/site/moffittcaspiprojects/home/concept-paper_2020/carlisi_2020a). Analyses were checked for reproducibility by an independent data analyst who recreated the code by working from the manuscript and applied it to a fresh copy of the data set.

## Results

Of the 938 Study members assessed at age 45 years, 875 were scanned. Of these 875, four were excluded due to major incidental findings or brain injuries, nine were excluded due to missing FLAIR scans, and one was excluded due to poor surface mapping, yielding 861 individuals for analysis. Of these, 9% (*N* = 80, 59% male) were previously phenotyped as exhibiting life-course-persistent antisocial behavior, 18% (*N* = 151, 54% male) as exhibiting adolescence-limited antisocial behavior, and 51% (*N* = 441, 47% male) as exhibiting low-antisocial behavior. These proportions resemble those in the full cohort, not all of whom underwent brain imaging (Odgers et al., 2008). Three Study members had missing information about their antisocial behavior, and the remaining 22% (*N* = 186) were identified as a childhood-limited antisocial group.

**Figure 1:**
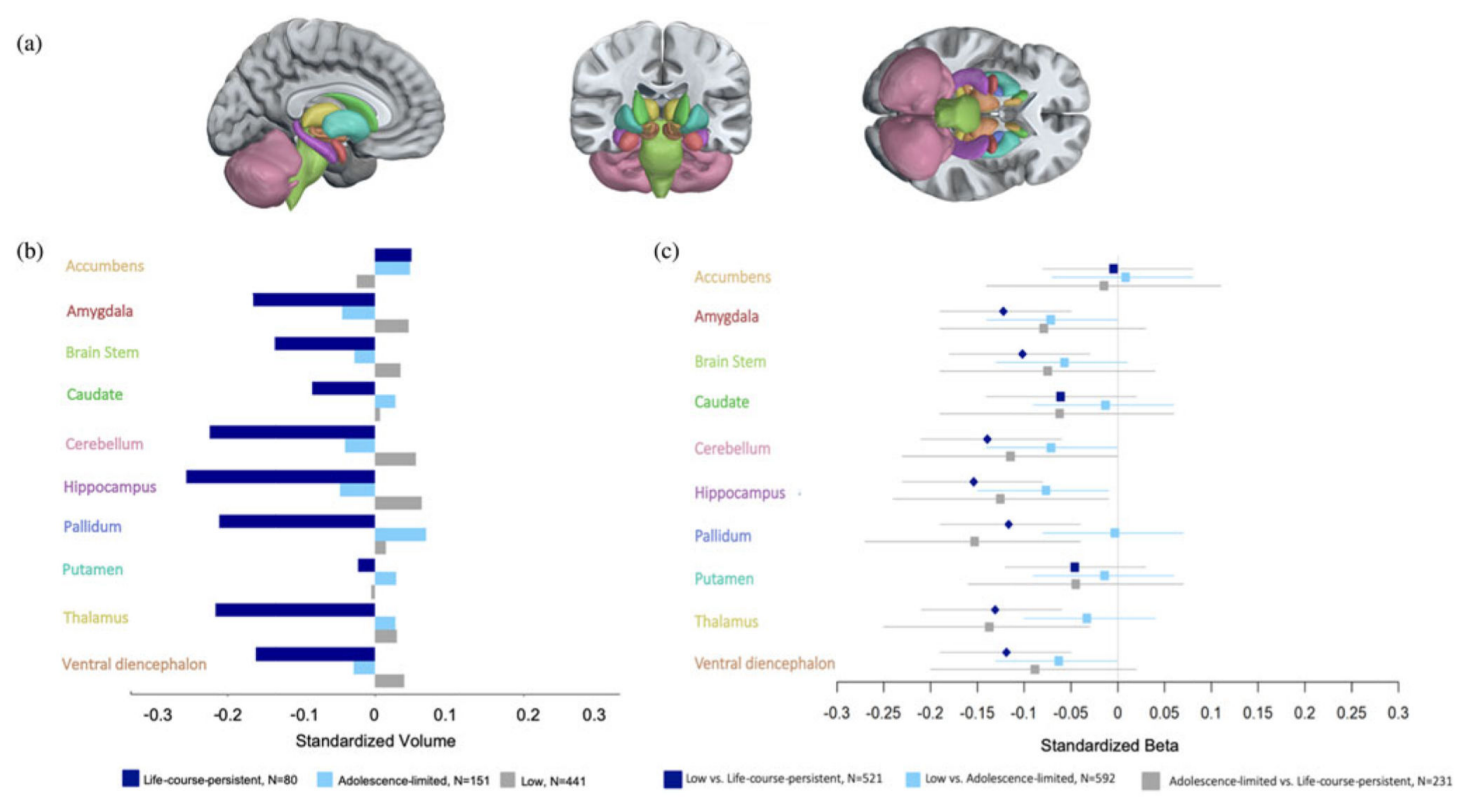
Comparisons of subcortical gray-matter volumes in life-course-persistent, adolescence-limited and low-antisocial groups. (a) Anatomical segmentation of subcortical regions of interest. (b) Plotted regional standardized mean volume by group. Data were standardized to a mean of zero and standard deviation of one. (c) Forest plot of regional effect sizes represented by standardized beta values and 95% confidence intervals for the comparison of each subcortical region of interest between the three antisocial groups. Diamonds indicate significance at *p* < .05 after FDR (false discovery rate) correction, whereas boxes indicate that the tests did not survive correction. Volumes are in units of mm^3^, standardized to *M* = 0, *SD* = 1. The Dunedin Study cohort is fully representative of its population so the zero point on a *z*-score from this sample equates to a population norm. The colors of the *y*-axis labels in (b) and (c) correspond with the colors of the brain regions in (a). Regions are listed alphabetically.

Our pre-registered primary research question was whether the absolute size of a brain region differs across groups. Figure 1 shows the segmentation of the subcortical structures investigated (Figure 1(a)), the mean standardized regional volumes for each group (Figure 1(b)), and the effect sizes associated with comparisons between the three groups (Figure 1(c)); also see Table 1). Effect sizes <0.2 are considered small, 0.2–0.4 moderate/large, and >0.4 very large (likely an overestimate) (Funder & Ozer, 2019). Three findings stand out. First, compared with the low-antisocial group, the life-course-persistent group had significantly lower gray-matter volume in the amygdala, brain stem, cerebellum, hippocampus, pallidum, thalamus, and ventral diencephalon. Second, there were no statistically significant differences in subcortical gray-matter volume between the adolescence-limited and low-antisocial groups. Actually, the mean volumes of subcortical structures in the adolescent-limited group broadly resembled those of the low-antisocial group, and were near the norm in this population-representative cohort (Figure 1(b)). Third, although regional gray-matter volume did not differ significantly between the adolescence-limited group and the life-course-persistent group, the statistical effect sizes for the differences between the life-course-persistent and adolescent-limited groups were similar to those between the life-course-persistent group and the low-antisocial group (Figure 1(c)): amygdala, −.12 and −.08; brain stem, −.10 and −.07; cerebellum, −.14 and −.11; hippocampus, −.15 and −.13; pallidum, −.12 and −.15; thalamus, −.13 and −.14; ventral diencephalon, −.12 and −.09 (these values showing the life-course-persistent vs. adolescence-limited effect size and the life-course-persistent vs. low-antisocial effect size, respectively). Comparisons between the adolescence-limited and the life-course-persistent groups with *N* = 231 did not attain statistical significance likely because they had less statistical power to detect group differences than the tests with *N* = 521 comparing the life-course-persistent and low-antisocial groups. The life-course-persistent group diverged to approximately the same extent from both the low-antisocial and adolescence-limited groups, who appeared most similar to one another, on subcortical gray-matter volume.

Life-course-persistent antisocial individuals grew up in more socioeconomically deprived families, were exposed to more adverse childhood experiences, had more head injuries, more impaired neuropsychological functioning, and higher rates of serious mental illness (schizophrenia) and drug/alcohol dependence (see Supplementary Table 1). Although these features were typically associated with the life-course-persistent phenotype and their effects on brain structure cannot be fully disentangled from those related uniquely to antisocial behavior, for the sake of completeness, secondary analyses evaluated whether these co-occurring features accounted for differences in subcortical gray-matter volume observed between individuals on the life-course-persistent antisocial trajectory and the other groups. Separate models were used to examine the effects of each covariate independently. All models controlled for sex. Exposure to deprivation/adversity, head injuries, serious mental illness, and drug/ alcohol dependence did not account for the observed differences between the groups (see Supplementary Tables 2–4). Differences in childhood IQ – a measure of neuropsychological functioning – statistically explained observed group differences in subcortical gray-matter volume, suggesting that the lower subcortical gray-matter volumes among individuals on the life-course-persistent trajectory also reflect their early-occurring neuropsychological deficits. Correlations among subcortical volumes, total brain volume, and global cortical measures are presented in Appendix 2 and Figure 2 of the Supplementary Material.

### Considering total brain volume

Supplementary Table 5 shows that life-course-persistent individuals had smaller mean total brain volume compared to low-antisocial (β = −0.14, 95% confidence interval (CI) [−0.21, −0.07], *p* < .0001) and adolescence-limited individuals (β = −0.12, 95% CI [−0.23, −0.12], *p* = .02). Adolescence-limited individuals also had smaller total brain volume compared to low-antisocial individuals (β = −0.06, 95% CI [−0.13, 0.00], *p* = .05), although the magnitude of this difference was considerably smaller than that of the life-course-persistent group comparisons. In consideration of these findings, particularly for the life-course-persistent group, we repeated our analyses of regional subcortical volumes, statistically controlling for total brain volume. This statistical control addressed the question of whether regional volumes differed between groups, beyond group differences in total brain volume. Regional differences between life-course-persistent and low-antisocial individuals were rendered statistically nonsignificant when controlling for total brain volume (Table 2). This indicates that the absolute regional volumes of subcortical structures that differ between groups are part of a nonspecific pattern of lower total brain volume in individuals with life-course-persistent antisocial behavior.

### Considering childhood-limited antisocial behavior

The “childhood-limited” trajectory was not hypothesized in the original developmental taxonomy theory (Moffitt, 1993), but subsequent studies have identified this group as a small group of children exhibiting extreme, pervasive, and persistent conduct problems only during childhood. Trajectory modeling of the Dunedin data has confirmed the existence of this group of individuals who exhibited elevated antisocial conduct near ages 5 and 7 years, apparently experiencing difficulty adjusting to school, but not at later ages (Odgers, Caspi et al., 2007). Like the life-course-persistent group, this group was distinguished in childhood by a history of maltreatment, low IQ, under-controlled temperament, and symptoms of attention-deficit/hyperactivity disorder (ADHD). However, unlike the life-course-persistent group, they did not come from families characterized by low socio-economic status, a parental criminal record or an elevated family history of psychiatric or substance abuse conditions (Odgers, Milne et al., 2007), and did not show abnormal scores on the polygenic score predictive of educational attainment (Wertz et al., 2018). This lack of familial risk factors discriminated them from the life-course-persistent group, who tended to have parents with crime records, a family history of psychiatric and substance conditions, and an elevated polygenic risk for poor educational attainment. The childhood-limited group, followed to adulthood, showed only low-level, intermittent criminal activity. When the Dunedin cohort was followed up to age 26 years, the members of this childhood-limited group, unlike other cohort individuals, were often social isolates; their informants reported that they had difficulty making friends, none had married, few held jobs, and many had diagnoses of agoraphobia and/or social phobia. As many as a third of this group had diagnosable depression, their personality profile showed elevated neuroticism, and their informants rated them as the most depressed, anxious individuals in the cohort (Moffitt, Caspi, Harrington, & Milne, 2002). This pattern of formerly antisocial individuals who appear to have recovered from early conduct problems and go on to develop into depressed, anxious, socially isolated individuals closely resembles a finding from a British longitudinal study of individuals followed from 8 to 32 years of age (Farrington, 1995). In that study, at-risk antisocial youths who became adult “false positives” (committing less crime than predicted) had few or no friends, held low-paid jobs, lived in unsanitary home conditions, and had been described in case records as withdrawn, highly strung, obsessional, nervous, or timid (Farrington, 1995).

**Figure 2:**
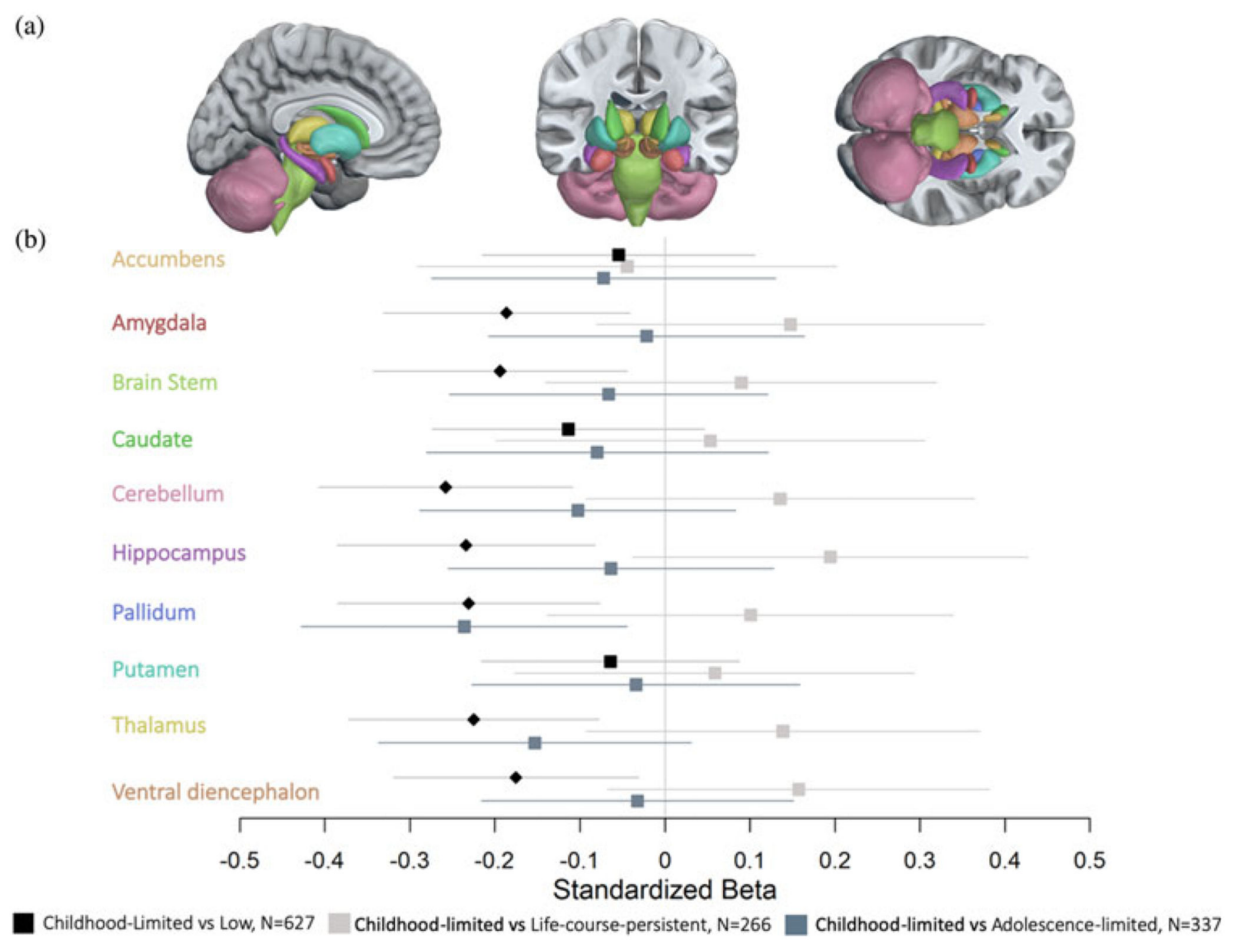
Comparisons of subcortical gray-matter volumes between the childhood-limited and life-course-persistent, adolescence-limited and low-antisocial groups. (a) Anatomical segmentation of subcortical regions of interest. (b) Forest plot of region-wise effect sizes represented by standardized beta values for the comparison of each subcortical region between the antisocial groups. All tests were corrected for multiple comparisons using false discovery rate (FDR). Diamonds indicate significance after FDR correction, whereas boxes indicate that the tests did not survive FDR correction. The colors of the *y*-axis labels correspond with the colors of the brain regions in (a). Subcortical regions represent areas that were significantly lower in cortical thickness in study members with childhood-limited antisocial behavior. All results are controlled for sex and FDR-corrected, *p* < .05. Regions are presented alphabetically. The analyses were performed in response to a reviewer request, not pre-registered.

An additional 186 Study members were classified as exhibiting childhood-limited antisocial behavior. Although not part of our pre-registered analyses, we also report findings for this group, which had a lower volume in seven of 10 subcortical structures when compared with the low-antisocial group (Table 3 and Figure 2). These differences overlapped with those observed when life-course-persistent individuals were compared with low-antisocial individuals. No statistically significant differences in subcortical volume were observed between the childhood-limited group and the adolescence-limited or life-course-persistent groups.

The overlap in regional findings between childhood-limited and life-course-persistent groups indicates that although a non-specific pattern of lower volume may be associated with life-course-persistent antisocial behavior, it is not associated with this outcome only. Based on existing evidence, both groups have a history of maltreatment, low childhood IQ, and under-controlled temperament as children, but the life-course-persistent group has additional quantitative risk factors of family histories of criminality, substance use, and psychiatric disorders, plus documented genetic risk for low educational attainment (Wertz et al., 2018). When considered in this context, the finding of similar structural brain differences between these groups suggests that while both have poor outcomes, individual (e.g., genetic) and contextual risk factors across the life span may lead to different expressions of the brain phenotype over time or determine the specific pattern of pathological adult behavior.

## Discussion

In a longitudinal study of a population-representative birth cohort, child to adult assessments of antisocial behavior and MRI data at age 45 years were used to compare subcortical gray-matter volumes in individuals characterized by life-course-persistent antisocial behavior, adolescence-limited antisocial behavior or low levels of antisocial behavior. Gray-matter volumes of subcortical structures broadly associated with affective and cognitive functions (including emotion regulation, goal-directed behavior, and decision making) and which have been previously implicated in studies of aggressive behavior (Goldin et al., 2008; Lamm et al., 2011; Shigemune et al., 2010) were lower in individuals on the life-course-persistent trajectory compared with individuals with a history of low-antisocial behavior. No significant differences were observed between the low-antisocial or the life-course-persistent group and individuals following the adolescence-limited antisocial trajectory, whose brains mostly resembled the normative brains of individuals with a history of low-antisocial behavior. However, effect size differences between the life-course-persistent and adolescent-limited groups were comparable to those seen in the comparison between the life-course-persistent and low-antisocial groups. Given the relatively small number of life-course-persistent individuals, the estimates of effect size provide useful information about the magnitude of group differences beyond statistical significance, which depends on sample size. These findings of subcortical differences are consistent with patterns observed in our previous study comparing the cortical anatomy of individuals following these trajectories of antisocial behavior (Carlisi et al., 2020).

Differences in subcortical gray-matter volumes between the life-course-persistent and low-antisocial groups appeared to overlap with differences in childhood neuropsychological functioning between these groups. This was confirmed in secondary comparisons of subcortical gray-matter volumes controlling for childhood IQ. In addition to lower total brain volume, lower cognitive ability was associated with life-course-persistent antisocial behavior (see Supplementary Table 1 (Wertz et al., 2018)). It was found that many of the control variables included in the supplementary analyses were implicated in the etiology and development of persistent antisocial behavior. Life-course-persistent individuals were characterized by both lower subcortical gray-matter volume as well as lower cognitive function, both of which may be under shared genetic influence or due to shared environmental exposures (e.g., maltreatment) (Blokland, de Zubicaray, McMahon, & Wright, 2012; McCrory, De Brito, & Viding, 2011). It could be argued that these factors helped to give rise to the observed differences in brain structure implicated in antisocial behavior. We present these secondary analyses for completeness and transparency, but inference drawn from the results of including these covariates should be carefully considered. Collectively, these results build a more comprehensive picture of the overall phenotype of life-course-persistent antisocial behavior and distinguish those on a life-course-persistent trajectory from those with adolescence-limited antisocial behavior.

This study has several strengths. First, developmental trajectories were defined by prospectively-tracked antisocial behavior from childhood to adulthood in a population-representative sample. Previous studies of brain differences associated with antisocial behavior have been carried out on relatively small samples, where age of onset was not assessed or was cross-sectionally defined by retrospective reports about antisocial behavior. Second, the individuals in our sample were unselected for treatment referral. As such, our study design provides a more accurate representation of the developmental taxonomy than previous studies and its results may help clarify inconsistencies in findings from previous studies of subcortical brain structure and antisocial behavior.

Lower amygdala and hippocampal gray-matter volume characterized the study members on the life-course-persistent trajectory, echoing findings from previous studies in youth with conduct disorders (Cope et al., 2014; Fairchild et al., 2011; Huebner et al., 2008; Sterzer, Stadler, Poustka, & Kleinschmidt, 2007). Our findings support that these associations typify individuals on a life-course-persistent rather than an adolescence-limited trajectory of antisocial behavior, paralleling results from a meta-analysis in individuals specifically with early-onset conduct disorder (Rogers & De Brito, 2016). A previous study found that men with higher levels of aggression from childhood to early adulthood (in line with the behavioral trajectory of life-course-persistent individuals) had lower amygdala gray-matter volume, which was associated with increased risk for aggression in adulthood (Pardini, Raine, Erickson, & Loeber, 2014). The amygdala is functionally connected with the hippocampus, brain stem, and hypothalamus, the gray-matter volumes of which were also lower in the life-course-persistent individuals. Animal and human studies have linked the hypothalamus and reduced hypothalamic-pituitary-adrenal axis function with aggression and emotion dysregulation, and it has been suggested that this link may be modulated by areas of the brain stem including the periaqueductal gray (Cappadocia, Desrocher, Pepler, & Schroeder, 2009; LeDoux, 2000). Moreover, a smaller hippocampus has been associated with aggressive behavior in adults (Zetzsche et al., 2007). However, alterations in these structures have been less consistently identified in studies of children with cross-sectionally defined early-onset or adolescent-onset conduct problems (Rogers & De Brito, 2016).

Lower cerebellar and thalamic gray-matter volume also characterized the life-course-persistent antisocial behavior study members. While the cerebellum is most commonly associated with motor functions, it also contributes to higher-order cognitive and emotional processes through connections within the cerebello-thalamo-cerebro-cortical circuits, where the cerebellum helps to compute internal models comparing action intention with execution and feedback (Romer et al., 2018). Moreover, lower cerebellar gray-matter volume has been identified as a trans-diagnostic feature of mental illness (Romer et al., 2018, 2019). Based on neurocognitive evidence of difficulties with decision making and goal-directed behavior in antisocial individuals (Moffitt, 2018) and that life-course-persistent Study members have elevated rates of psychiatric disorders (Carlisi et al., 2020), the lower cerebellar and thalamic volumes observed in life-course-persistent individuals may reflect impaired information processing necessary to adaptively shape actions and desist from antisocial behavior.

No differences between the comparison groups were observed in accumbens (i.e., ventral striatum), caudate or putamen (i.e., dorsal striatum) volumes. This finding was unexpected given (a) the ventral striatumߣs central role in prediction error and expected value, which are disrupted in children and adults with antisocial behavior (Blair, 2016), and (b) the dorsal striatum’s role in reinforcement learning, which is characteristically impaired in antisocial individuals, in line with their reduced ability to compute the consequences of their actions (O’Doherty et al., 2004). Our prior study found cortical differences in ventromedial prefrontal and orbitofrontal regions in individuals on the life-course-persistent trajectory (Carlisi et al., 2020). Given that these cortical regions are interconnected with both the dorsal striatum and ventral striatum (O’Doherty et al., 2004), taken together, these findings may help to resolve discrepancies within the literature regarding striatal abnormalities in antisocial behavior; the reward-related behavioral abnormalities associated with persistent antisocial behavior may be a result of disruption in top-down cortical regulation of the striatum rather than within specific subregions of the striatum itself, particularly in the most extreme examples of externalizing behavior. However, it is difficult to equate structure with function – functional neuroimaging analyses of striatal subregions in anti-social trajectory groups will be informative.

This study has limitations. First, we acknowledge that because structural brain differences were assessed at 45 years of age, we were unable to determine whether these features relate to genetic or early life risk factors that might lead to an antisocial lifestyle or whether they are a consequence of a persistent antisocial lifestyle. It is possible that differences were present from early childhood, before the onset of antisocial behavior, and played a causal role in the development of such behavior. It is equally plausible that the observed group differences in volume are a result of group differences in features associated with the complex phenotype of antisocial behavior (although covariate analyses showed that low socioeconomic status, adversity, drugs/alcohol, head injuries, or serious mental illness did not account for group differences). Risk factors in life-course-persistent individuals that are under partial genetic influence, such as low childhood cognitive ability, suggest that some of the brain differences observed in the present study could have been present from early life. In this regard, the Dunedin Study previously reported that the life-course-persistent group, compared with the adolescence-limited and low-antisocial groups, had a significantly lower mean educational attainment polygenic score, which is thought to reflect emerging traits such as low cognitive ability and poor self-control that increase the risk of early antisocial behavior (Wertz et al., 2018). However, given the comparatively recent advent of MRI (and the capacity to apply it in large nonclinical samples), no study combines data prospectively tracking antisocial behavior into adulthood with brain scans taken in early childhood. Second, the Dunedin Study members were born in one part of the world, potentially limiting generalizability. Third, while we were able to ensure that our findings about associations between brain structure and antisocial behavior were not confounded by sex differences, we were not sufficiently powered to test sex differences in these associations (Moffitt & Caspi, 2001; Odgers et al., 2008). Large studies that include both males and females are needed to explore this issue. The Dunedin Study is a well-established cohort representative of the general population, but our results should be replicated in other samples prospectively followed across development. Finally, visual inspection of the analyses comparing subcortical gray-matter volumes between the adolescence-limited and low-antisocial groups revealed that the confidence intervals for the amygdala, cerebellum, hippocampus, and ventral diencephalon did not overlap with zero (Figure 1), which suggests that these regions may be implicated in the adolescence-limited trajectory. However, as the effect sizes of these differences were generally very small, did not reach statistical significance, and were not hypothesized a priori, future research is needed to determine if such differences are consistently identified between these trajectories and, if so, how they may shape the emergence of adolescence-limited antisocial behavior.

## Conclusion

The Developmental Taxonomy Theory of Antisocial Behavior has influenced both clinical practice and juvenile justice policy (Moffitt, 2018), prompting the recognition of heterogeneity among individuals who are diagnosed with conduct disorder or who appear before the criminal courts. Consistent with the taxonomy, we observed subcortical gray-matter volume differences among individuals following a life-course-persistent trajectory of antisocial behavior but not among individuals on the adolescence-limited trajectory. The small number of individuals whose antisocial behavior arises early and persists into midlife tend to have a brain structure that is distinct from the larger majority of antisocial individuals who present a lower risk of persisting with antisocial behavior and who are generally capable of reform. Our findings about subcortical structural differences between individuals on the adolescence-limited versus life-course-persistent trajectories, together with recent findings of cortical differences (Carlisi et al., 2020) and genetic risk differences (Wertz et al., 2018), validate the developmental taxonomy by providing new evidence that all antisocial individuals are not the same. Individuals on the life-course-persistent trajectory are characterized by correlated brain and behavior features. It is not the case that any one of these features (e.g., total brain volume, subcortical gray-matter volumes, IQ) “accounts for” or “confounds” the other. Rather, they all contribute to a phenotype characterizing the most severe and persistent antisocial individuals in society. This evidence underscores the need for greater developmental precision in studying conduct disorder and greater diagnostic precision in treating it. Of note, neuroimaging and genetic information is not sufficiently advanced to discriminate between different developmental trajectories of antisocial behavior (Moffitt, 2018), and practitioners working with families and individuals will generally not have access to prospective longitudinal data in order to distinguish between individuals following different trajectories. Instead, clinicians can leverage other reliable information to aid evidence-based prediction of prognostic trajectory, including family history of externalizing disorders, comorbid conduct disorder/ADHD, low IQ, low family social status, maltreatment history, and callous-unemotional traits (Moffitt, 2018; Odgers, Milne et al., 2007).

## Supplementary Material

The supplementary material for this article can be found at https://doi.org/10.1017/S0954579421000377

Supplementary Information

## Figures and Tables

**Table 1:** Mean subcortical gray-matter volumes and comparisons of individuals classified as having life-course persistent, adolescence-limited and low-antisocial behavior trajectories

	Raw means (*SD*)			Standardized scores, *M* (*SD*); *M* = 0, *SD* = 1			Comparison between trajectory groups (*β*, [95% CI], *p*-value)		
	Life-course-persistent (*N* = 80)	Adolescence-limited (*N* = 151)	Low (*N* = 441)	Life-course-persistent	Adolescence-limited	Low	Life-course-persistent versus low	Adolescence-limited versus low	Life-course-persistent versus adolescence-limited
Accumbens	493 (67)	493 (76)	487 (77)	.05 (.88)	.05 (1.00)	–.02 (1.02)	.00, [–0.08, 0.08], *p* = .91	.01, [–0.07, 0.08], *p* = .92	–.01, [–0.14, 0.11], *p* = .81
Amygdala	1,710 (191)	1,735 (219)	1,753 (204)	–.16 (.92)	–.04 (1.06)	.05 (.99)	–.12, [–0.19, –0.05], ***p* = .002**	–.07, [–0.14, 0.00], *p* = .16	–.08, [–0.19, 0.03], *p* = .27
Brain stem	21,491 (2,313)	21,770 (2,581)	21,931 (2,653)	–.14 (.89)	–.03 (.99)	.03 (1.02)	–.10, [–0.18, –0.03], ***p* = .01**	–.06, [–0.13, 0.01], *p* = .23	–.07, [–0.19, 0.04], *p* = .27
Caudate	3,286 (465)	3,333 (449)	3,324 (403)	–.09 (1.10)	.03 (1.07)	.01 (.96)	–.06, [–0.14, 0.02], *p* = .18	–.01, [–0.09, 0.06], *p* = .91	–.06, [–0.19, 0.06], *p* = .41
Cerebellum	59,287 (5,931)	60,442 (5,962)	61,044 (6,465)	–.22 (.94)	–.04 (.94)	.05 (1.02)	–.14, [–0.21, –0.06], ***p* = .002**	–.07, [–0.14, 0.00], *p* = .17	–.11, [–0.23, 0.00], *p* = .11
Hippocampus	4,227 (382)	4,315 (416)	4,362 (433)	–.26 (.90)	–.05 (.98)	.06 (1.02)	–.15, [–0.23, –0.08], ***p* < .001**	–.08, [–0.15, –0.01], *p* = .17	–.13, [–0.24, –0.01], *p* = .09
Pallidum	1,939 (220)	2,001 (216)	1,989 (221)	–.21 (1.00)	.07 (.98)	.01 (1.00)	–.12, [–0.19, –0.04], ***p* = .006**	.00, [–0.08, 0.07], *p* = .93	–.15, [–0.27, –0.04], *p* = .08
Putamen	4,692 (521)	4,719 (509)	4,701 (545)	–.02 (.98)	.03 (.95)	–.01 (1.02)	–.05, [–0.12, 0.03], *p* = .26	–.01, [–0.09, 0.06], *p* = .91	–.04, [–0.16, 0.07], *p* = .50
Thalamus	7,639 (793)	7,835 (813)	7,837 (801)	–.22 (.99)	.03 (1.01)	.03 (1.00)	–.13, [–0.21, –0.06], ***p* = .002**	–.03, [–0.10, 0.04], *p* = .58	–.14, [–0.25, –0.03], *p* = .08
Ventral diencephalon	4,084 (356)	4,139 (430)	4,167 (422)	–.16 (.85)	–.03 (1.03)	.04 (1.01)	–.12, [–0.19, –0.05], ***p* = .002**	–.06, [–0.13, 0.00], *p* = .17	–.09, [–0.20, 0.02], *p* = .21

All volumes in the left-hand section of the table are in units of mm^3^. The standardized means for each region were calculated to facilitate comparisons of group differences across the different regions. Standardized scores (middle section of the table) were set to a mean of 0 and standard deviation of 1. All analyses controlled for sex and group comparisons were corrected for multiple testing using a false discovery rate (FDR) procedure. *SD* = standard deviation. CI = confidence interval. *p*-values for significant differences (*p* < .05, FDR-corrected) between groups are presented in bold font. The regression coefficients (β values) are standardized coefficients.

**Table 2:** Comparisons of regional subcortical gray-matter volumes between trajectory groups, controlling for total brain volume

	β, 95% CI, *p*-value		
	Life-course-persistent versus low-antisocial	Adolescence-limited versus low-antisocial	Life-course-persistent versus adolescence-limited
Accumbens	0.08, [0.01, 0.05], *p* = .18	0.05, [–0.02, 0.11], *p* = .51	.05, [–0.06, 0.16], *p* = .76
Amygdala	–0.04, [–0.09, 0.02], *p* = .61	–0.03 [–0.09, 0.02], *p* = .51	–.01 [–0.10, 0.09], *p* = .90
Brain stem	0.01 [–0.05, 0.06], *p* = .81	.03 [–0.06, 0.04], *p* = .73	.02, [–0.06, 0.10], *p* = .84
Caudate	0.02, [–0.05, 0.09], *p* = .68	.03, [–0.04, 0.09], *p* = .55	.02, [–0.08, 0.13], *p* = .84
Cerebellum	–0.06, [–0.13, 0.00], *p* = .23	.03, [–0.10, 0.03], *p* = .51	–.05, [–0.14, 0.05], *p* = .76
Hippocampus	–0.07, [–0.13, –0.01], *p* = .18	.03, [–0.10, 0.02], *p* = .51	–.05, [–0.14, 0.04], *p* = .76
Pallidum	–0.02, [–0.08, 0.04], *p* = .68	.03, [–0.02, 0.10], *p* = .51	–.07, [–0.17, 0.02], *p* = .76
Putamen	0.03, [–0.03, 0.10], *p* = .61	.03, [–0.04, 0.08], *p* = .55	.03, [–0.07, 0.13], *p* = .84
Thalamus	–0.01, [–0.06, 0.03], *p* = .68	.02, [–0.02, 0.06], *p* = .55	–.03, [–0.10, 0.04], *p* = .76
Ventral diencephalon	–0.01, [–0.06, 0.04], *p* = .68	.02, [–0.06, 0.03], *p* = .59	.01, [–0.06, 0.08], *p* = .90

All analyses were controlled for sex and total brain volume. Group comparisons were corrected for multiple testing using a false discovery rate (FDR) procedure. CI =confidence interval. Regression coefficients (β values) are standardized coefficients. Analyses were performed in response to a reviewer request, not pre-registered.

**Table 3:** Mean subcortical gray-matter volumes for the childhood-limited group and group comparisons

		Comparisons between trajectory groups (β, 95% CI, *p*-value)		
	Childhood-limited (*N* =186), *M* (*SD*)	Childhood-limited versus Low	Childhood-limited versus Life-course-persistent	Childhood-limited versus Adolescence-limited
Accumbens	486 (84)	–.02, [–0.10, 0.05], *p* = .51	–.02, [–0.13, 0.09], *p* = .72	–.04, [–0.14, 0.06], *p* = .74
Amygdala	1,727 (198)	–.09, [–0.15, –0.02], ***p* = .02**	.07, [–0.04, 0.1], *p* = .49	–.01, [–0.10, 0.08], *p* = .82
Brain stem	21,564 (2,436)	–.09, [–0.16, –0.02], ***p* = .02**	.04, [–0.06, 0.15], *p* = .64	–.03, [–0.13, 0.06], *p* = .74
Caudate	3,294 (416)	–.05, [–0.13, 0.02], *p* = .21	.02, [–0.09, 0.14], *p* = .72	–.04, [–0.14, 0.06], *p* = .74
Cerebellum	59,733 (5,974)	–.12, [–0.19, –0.05], ***p* = .01**	.06, [–0.04, 0.17], *p* = .49	–.05, [–0.14, 0.04], *p* = .74
Hippocampus	4,283 (414)	–.11 (-.18, –0.04], ***p* = .01**	.09, [–0.02, 0.20], *p* = .49	–.03, [–0.13, 0.06], *p* = .74
Pallidum	1,949 (196)	–.11, [–0.18, –0.03], ***p* = .01**	.05, [–0.06, 0.16], *p* = .64	–.12, [–0.21, –0.02], *p* = .16
Putamen	4,694 (545)	–.03, [–0.10, 0.04], *p* = .45	.03, [–0.08, 0.13], *p* = .72	–.02, [–0.11, 0.08], *p* = .81
Thalamus	7,701 (763)	–.1, [–0.17, –0.04], ***p* = .01**	.06, [–0.04, 0.17], *p* = .49	–.08, [–0.17, 0.02], *p* = .52
Ventral diencephalon	4,119 (381)	–.08, [–0.15, –0.01], ***p* = .03**	.07, [–0.03, 0.18], *p* = .49	–.02, [–0.11, 0.07], *p* = .81

All volumes in the left-hand section of the table are reported in units of mm^3^. All analyses were controlled for sex and group comparisons were corrected for multiple testing using a false discovery rate (FDR) procedure. Results surviving FDR correction (*p* < .05) are shown in bold font. *SD* = standard deviation. CI = confidence interval. Regression coefficients (β values) are standardized coefficients. Analyses were performed in response a to reviewer request, not pre-registered.

## Data Availability

The data sets reported in the current article are available to qualified scientists on request. Such requests require a concept paper describing the purpose of data access, ethical approval at the applicant’s university, and provision for secure data access (https://moffittcaspi.trinity.duke.edu/research). We offer secure access on the campuses of Duke University (Durham, NC, USA), University of Otago (Dunedin, New Zealand), and King’s College London (London, UK). All data analysis scripts and results files are available for review.
