# Supplementary material for: Association of subcortical gray-matter volumes with life-course-persistent antisocial behavior in a population-representative longitudinal birth cohort
Source: Dev Psychopathol. Author manuscript; Available in PMC 2022 Dec 23. (PMC7613992; doi:10.1017/S0954579421000377)
Supplement: Supplementary Information [file EMS145880-supplement-Supplementary_Information.pdf]

Supplementary Information

Association of subcortical grey-matter volumes with life-course-persistent antisocial behavior in a population-representative longitudinal birth cohort

CONTENTS

eAppendix 1. Attrition analysis……………………………………………………….…………………...2

eFigure 1. FreeSurfer aseg results overlaid on one Dunedin Study member's T1-weighted structural scan……..3

eTable 1. Demographic, cognitive and psychiatric characteristics…………………….……………………….4

eTable 2. Implications of statistically controlling for co-occurring experiences and conditions on comparisons of subcortical grey-matter volume between life-course-persistent and low-antisocial trajectory groups.………..…5

eTable 3. Implications of statistically controlling for co-occurring experiences and conditions on comparisons of subcortical grey-matter volume between adolescence-limited and low-antisocial trajectory groups………..……………………………………………………………………………………......…6

eTable 4. Implications of statistically controlling for co-occurring experiences and conditions on comparisons of subcortical grey-matter volume between life-course-persistent and adolescence-limited trajectory groups……...7

eTable 5. Average total brain volume and antisocial group comparisons………………………………………...8

eAppendix 2. Relationships among global and regional subcortical variables…………………………..……..9

eFigure 2. Correlations among regional subcortical grey-matter volumes and total brain volume, total surface area and average cortical thickness………………………………………………………………….…......9

***eAppendix 1.* Attrition analysis**

An attrition analysis was conducted using childhood socioeconomic status (SES) and childhood IQ to determine whether participants in the age-45 data collection were representative of the original cohort.

No significant differences were found between the full cohort, those deceased, those alive, those seen at age 45 or those scanned at age 45 on childhood SES.

Attrition analysis of childhood SES comparing age-45 study members with original cohort

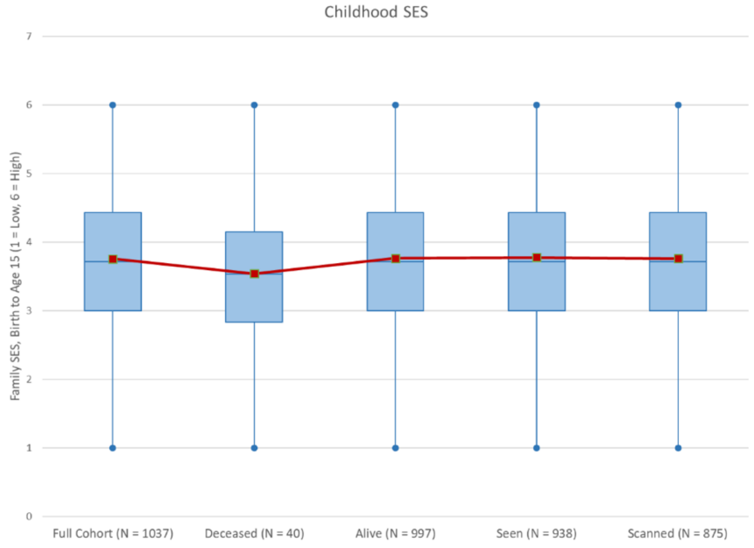

No significant differences in childhood IQ were found between the full cohort, those still alive, those seen at age-45 or those scanned at age-45. Those who were deceased by the age-45 data collection had significantly lower childhood IQ's than those who were still alive (t=2.09, p=0.04).

Attrition analysis of childhood IQ comparing age-45 study members with original cohort

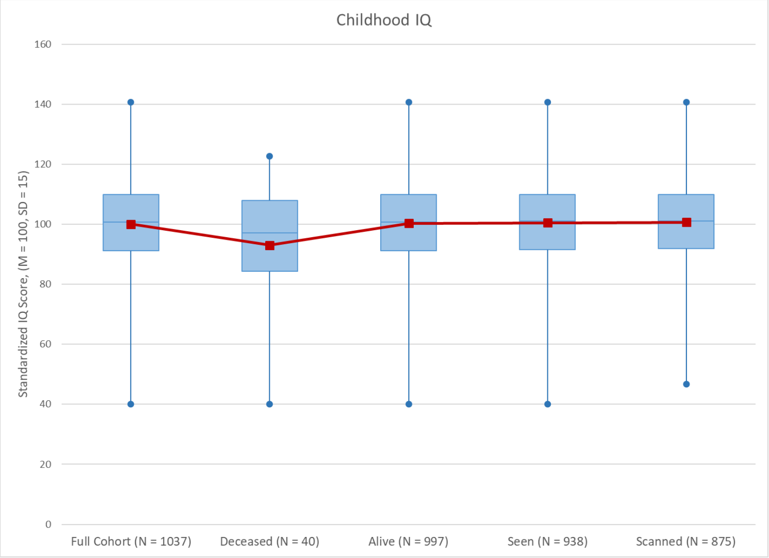

eFigure 1. FreeSurfer aseg results overlaid on one Dunedin Study member's T1-weighted structural scan

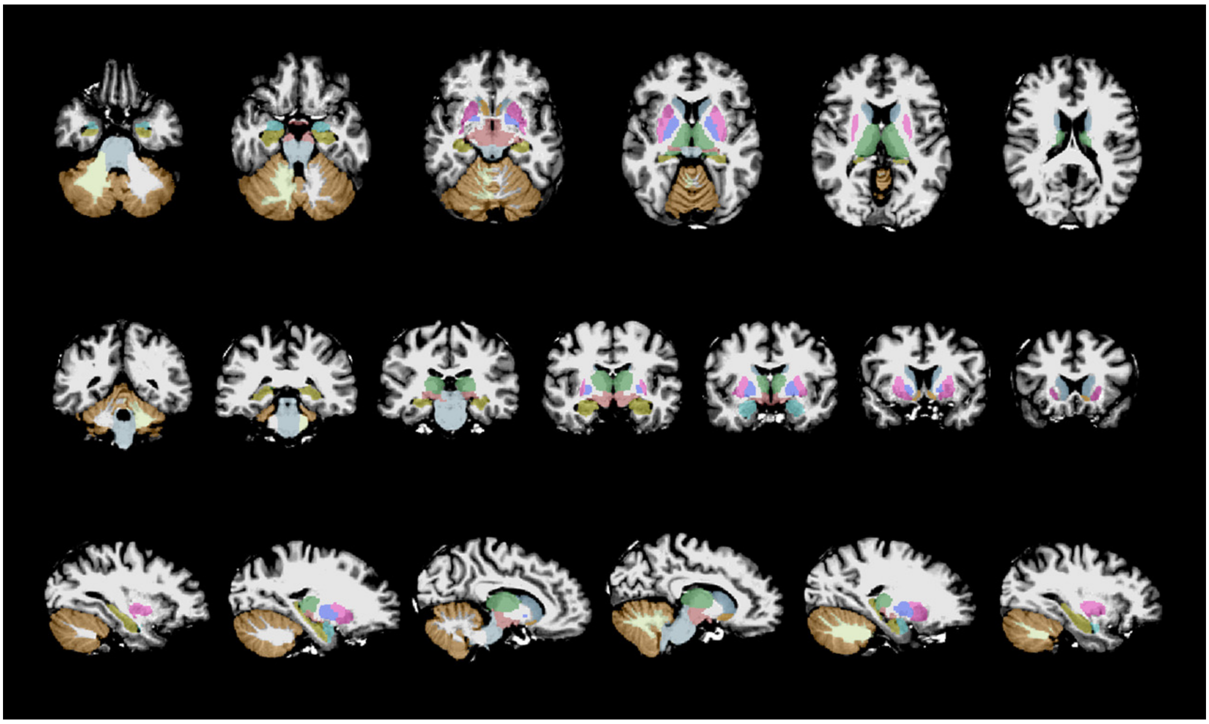

eTable 1. Demographic characteristics and co-occurring experiences and conditions associated with antisocial behavior. All measures are described in the main text.

	Antisocial trajectory groups[a]			*Comparisons between trajectory groups*		
	**Life-course-persistent**	**Adolescence-limited**	**Low**	**Life-course-persistent vs Low**	**Adolescence-limited vs Low**	**Life-course-persistent vs Adolescence-limited**
**N**	80	151	441	..	..	..
**% male (N)**	58.75 (47)	54.30 (82)	47.39 (209)	OR = 1.58 (0.98-2.56)	OR = 1.32 (0.91-1.91)	OR=1.20 (0.69-2.07)
**Childhood SES (M, SD)**[b]	3.03 (0.98)	3.58 (1.08)	4.03 (1.12)	7.51 (515) p < .001	4.31 (585) p < .001	3.80 (228) p < .001
**ACEs, M (SD)**[c]	2.125 (1.50)	1.113 (1.18)	0.739 (.95)	10.81 (519) p < .0001	3.91 (590) p = .0001	5.64 (229) p < .0001
**Childhood WISC-R IQ, M (SD)**[d]	93.24 (13.70)	101.21 (14.00)	103.81 (13.10)	6.55 (513) p < .0001	2.06 (584) p = .0395	4.12 (227) p < .0001
**Head injury, % (N)**	11.25 (9)	18.54 (28)	9.98 (44)	OR = 1.14 (0.54-2.45)	OR = 2.05 (1.23-3.44)	OR=.56 (0.25-1.25)
**Schizophrenia, % (N)**	11.25 (9)	6.62 (10)	0.45 (2)	OR=27.82 (5.89-131.43)	OR=15.57(3.37-71.89)	OR=1.79 (0.70-4.60)
**Drug/Alcohol dependence, % (N)**	32.50 (26)	23.84 (36)	9.55 (42)	OR=4.56 (2.59-8.03)	OR = 2.97 (1.82-4.85)	OR=1.54 (0.85-2.80)

A similar version of this table including the same groups of Study members is presented in Carlisi et al., 2020

Values for between-group comparisons are reported as odds ratios (OR) (95% confidence intervals), or t-statistics (degrees of freedom), p-values.

[a] Growth-mixture modelling was applied to derive developmental subtypes of antisocial behavior, within sex, as previously published in Carlisi et al., 2020

[b] SES**,** socio-economic status. The scale ranges from 1 (low) to 6 (high).

[c] ACE, adverse childhood experience. The scale ranges from 0 (none) to 4+.

eTable 2. Implications of statistically controlling for co-occurring experiences and conditions on comparisons of subcortical grey-matter volume between life-course-persistent and low-antisocial trajectory groups

	Life-course-persistent vs. low-antisocial [β (95% CI), p-value]						
	**Sex only**	**Childhood SES**	**ACEs**	**Childhood IQ**	**Head Injury**	**Schizophrenia**	**Drug/Alcohol Dependence**
Accumbens	.00 (-.08-.08) p=.91	-.04 (-.13-.05) p=.48	.00 (-.08-.08) p=.95	.03 (-.05-.01) p=.57	0.00 (-.08-.08) p=.92	-.01 (-.09-.07) p=.87	0.00 (-.08-.08) p=.96
Amygdala	-.12 (-.19--.05) **p=.002**	-.10 (-.18--.02) **p=.04**	-.11 (-.18--.04) **p=.02**	-.08 (-.15-0.00) p=.12	-.12 (-.19--.05) **p=.002**	-.12 (-.19--.05) **p=.006**	-.12 (-.20--.05) **p=.003**
Brain Stem	-.10 (-.18--.03) **p=.01**	-.10 (-.18--.02) **p=.04**	-.09 (-.16--.01) **p<.05**	-.04 (-.12-.03) p=.42	-.10 (-.18--.03) **p=.01**	-.09 (-.16--.03) **p=.04**	-.11 (-.18--.03) **p=.009**
Caudate	-.06 (-.14-.02) p=.18	-.02 (-.11-.07) p=.64	-.04 (-.13-.04) p=.44	-.02 (-.10-.06) p=.76	-.06 (-.14-.02) p=.17	-.05 (-.13-.03) p=.34	-.08 (-.16-.00) p=.08
Cerebellum	-.14 (-.21--.06) **p=.002**	-.13 (-.21--.04) **p=.02**	-.12 (-.19--.04) **p=.02**	-.08 (-.15--.01) p=.12	-.14 (-.21--.06) **p=.002**	-.13 (-.20--.05) **p=.006**	-.15 (-.23--.08) **p<.001**
Hippocampus	-.15 (-.23--.08) **p < .001**	-.13 (-.21--.05) **p=.02**	-.14 (-.21--.06) **p=.01**	-.12 (-.19--.04) **p=.03**	-.15 (-.23--.08) **p=.0006**	-.16 (-.24--.09) **p=.0005**	-.15 (-.22--.07) **p<.001**
Pallidum	-.12 (-.19--.04) **p=.006**	-.09 (-.17-0.00) p=.08	-.11 (-.19--.04) **p=.02**	-.08 (-.16-0.00) p=.12	-.12 (-.19--.04) **p=.005**	-.10 (-.18--.02) **p=.03**	-.12 (-.20--0.4) **p=.006**
Putamen	-.05 (-.12-.03) p=.26	-.04 (-.12-.04) p=.47	-.04 (-.11-.04) p=.44	-.01 (-.08-.07) p=.88	-.05 (-.12-.03) p=.26	-.04 (-.12-.03) p=.34	-.05 (-.12-.03) p=.26
Thalamus	-.13 (-.21--.06) **p=.002**	-.13 (-.21--.05) **p=.02**	-.11 (-.18--.03) **p=.02**	-.07 (-.15-0.00) p=.12	-.13 (-.21--.06) **p=.002**	-.11 (-.18--.04) **p=.01**	-.13 (-.20--.06) **p=.003**
Ventral Diencephalon	-.12 (-.19--.05) **p=.002**	-.11 (-.18-.03) **p=.03**	-.10 (-.17--.02) **p=.03**	-.07 (-.14-0.00) p=.12	-.12 (-.19--.05) **p=.002**	-.10 (-.18--.03) **p=.01**	-.11 (-.18--.04) **p=.006**

All analyses controlled for sex, and group comparisons were corrected for multiple testing using a false discovery rate (FDR) procedure. Results in the first column, "sex only", are those presented in the main text, Table 1. *P*-values for significant differences (p<.05, FDR corrected) between groups are presented in **bold.** ACEs = adverse childhood experiences, CI = confidence interval, SES = socioeconomic status. Regression coefficients (betas) are standardized coefficients.

eTable 3. Implications of statistically controlling for co-occurring experiences and conditions on comparisons of subcortical grey-matter volume between adolescence-limited and low-antisocial trajectory groups

	Adolescence-limited vs. low-antisocial [β (95% CI), p-value]						
	**Sex only**	**Childhood SES**	**ACEs**	**Childhood IQ**	**Head Injury**	**Schizophrenia**	**Drug/Alcohol Dependence**
Accumbens	.01 (-.07-.08) p=.92	-.02 (-.10-.06) p=.78	.02 (-.06-.09) p=.97	.02 (-.06-.09) p=.97	.01 (-.06-.09) p=.89	.02 (-.05-.10) p=.83	.02 (-.06-.09) p=.72
Amygdala	-.07 (-.14-.00) p=.16	-.06 (-.13-.02) p=.30	-.06 (-.13-.01) p=.34	-.06 (-.12-.01) p=.38	-.07 (-.14--.01) p=.19	-.07 (-.13-0.00) p=.25	-.07 (-.13-.00) p=.21
Brain Stem	-.06 (-.13-.01) p=.23	-.06 (-.14-.01) p=.30	-.04 (-.11-.03) p=.46	-.04 (-.11-.03) p=.52	-.05 (-.12-.02) p=.30	-.05 (-.12-.02) p=.43	-.06 (-.13-.01) p=.24
Caudate	-.01 (-.09-.06) p=.91	-.02 (-.10-.06) p=.78	0.00 (-.08-.07) p=.97	0.00 (-.07-.07) p=.99	-.01 (-.09-.06) p=.89	-.01 (-.09-.06) p=.84	-.02 (-.10-.05) p=.72
Cerebellum	-.07 (-.14-.00) p=.17	-.06 (-.14-.01) p=.30	-.06 (-.13-.01) p=.34	-.05 (-.12-.02) p=.38	-.07 (-.14-0.00) p=.21	-.07 (-.14-.01) p=.25	-.07 (-.14-.00) p=.21
Hippocampus	-.08 (-.15--.01) p=.17	-.07 (-.15-0.00) p=.30	-.07 (-.14-0.00) p=.34	-.06 (-.13-.01) p=.38	-.08 (-.15-0.00) p=.19	-.08 (-.15--.01) p=.25	-.07 (-.14-.00) p=.21
Pallidum	.00 (-.08-.07) p=.93	.01 (-.06-.09) p=.79	.01 (-.06-.08) p=.97	.01 (-.06-.08) p=.99	0.00 (-.07-.07) p=.99	-.01 (-.08-.07) p=.85	-.01 (-.08-.07) p=.84
Putamen	-.01 (-.09-.06) p=.91	0.00 (-.08-.07) p=.93	0.00 (-.08-.07) p=.97	0.00 (-.07-.07) p=.99	-.01 (-.08-.06) p=.89	-.01 (-.08-.06) p=.84	-.02 (-.09-.05) p=.72
Thalamus	-.03 (-.10-.04) p=.58	-.03 (-.11-.04) p=.61	-.02 (-.09-.05) p=.96	-.02 (-.09-.05) p=.94	-.03 (-.10-.04) p=.69	-.02 (-.09-.05) p=.83	-.03 (-.10-.04) p=.72
Ventral Diencephalon	-.06 (-.13-.00) p=.17	-.05 (-.13-.02) p=.30	-.05 (-.12-.02) p=.39	-.05 (-.11-.02) p=.38	-.06 (-.13-.01) p=.23	-.05 (-.12-.02) p=.35	-.05 (-.12-.01) p=.24

All analyses controlled for sex, and group comparisons were corrected for multiple testing using a false discovery rate (FDR) procedure. Results in the first column, "sex only", are those presented in the main text, Table 1. *P*-values for significant differences ($p<.05$, FDR corrected) between groups are presented in **bold.** ACEs = adverse childhood experiences, CI = confidence interval, SES = socioeconomic status. Regression coefficients (betas) are standardized coefficients.

eTable 4. Implications of statistically controlling for co-occurring experiences and conditions on comparisons of subcortical grey-matter volume between life-course-persistent and adolescence-limited trajectory groups

	Life-course-persistent vs. adolescence-limited [β (95% CI), p-value]						
	**Sex only**	**Childhood SES**	**ACEs**	**Childhood IQ**	**Head Injury**	**Schizophrenia**	**Drug/Alcohol Dependence**
Accumbens	-.01 (-.14-.11) p=.81	-.02 (-.16-.12) p=.75	.02 (-.10-.14) p=.89	.06 (-.06-.17) p=.88	-.02 (-.14-.10) p=.75	-.01 (-.13-.11) p=.88	-.02 (-.14-.10) p=.80
Amygdala	-.08 (-.19-.03) p=.27	-.14 (-.27-0.00) p=.13	-.08 (-.19-.03) p=.41	-.01 (-.12-.09) p=.96	-.08 (-.19-.03) p=.25	-.08 (-.19-.03) p=.29	-.08 (-.19-.03) p=.20
Brain Stem	-.07 (-.19-.04) p=.27	-.11 (-.25-.03) p=.17	-.06 (-.17-.05) p=.46	0.00 (-.11-.11) p=.96	-.08 (-.19-.03) p=.25	-.07 (-.18-.04) p=.31	-.08 (-.20-.03) p=.20
Caudate	-.06 (-.19-.06) p=.41	-.12 (-.26-.03) p=.17	.02 (-.11-.14) p=.89	.01 (-.11-.13) p=.96	-.06 (-.19-.06) p=.41	-.06 (-.19-.06) p=.40	-.08 (-.2-.05) p=.29
Cerebellum	-.11 (-.23-.00) p=.11	-.10 (-.24-.04) p=.21	-.09 (-.20-.02) p=.41	-.04 (-.15-.06) p=.88	-.12 (-.23--.01) p=.09	-.11 (-.22-0.00) p=.12	-.13 (-.24--.02) p=.06
Hippocampus	-.13 (-.24--.01) p=.09	-.18 (-.31--.04) **p=.04**	-.12 (-.24--.01) p=.41	-.04 (-.15-.06) p=.88	-.13 (-.24--.02) p=.07	-.12 (-.24--.01) p=.11	-.13 (-.24--.02) p=.06
Pallidum	-.15 (-.27--.04) p=.08	-.18 (-.31--.04) **p=.04**	-.08 (-.20-.03) p=.41	-.09 (-.21-.02) p=.88	-.16 (-.28--.04) p=.07	-.15 (-.27--.03) p=.11	-.16 (-.28--.05) p=.06
Putamen	-.04 (-.16-.07) p=.50	-.05 (-.18-.09) p=.56	.01 (-.11-.12) p=.89	.02 (-.09-.14) p=.96	-.05 (-.17-.06) p=.41	-.04 (-.16-.08) p=.55	-.05 (-.17-.06) p=.40
Thalamus	-.14 (-.25--.03) p=.08	-.15 (-.28--.02) p=.07	-.10 (-.21-.02) p=.41	-.04 (-.14-.06) p=.88	-.14 (-.25--.03) p=.07	-.13 (-.24--.01) p=.11	-.14 (-.26--.03) p=.06
Ventral Diencephalon	-.09 (-.20-.02) p=.21	-.11 (-.24-.01) p=.16	-.06 (-.17-.05) p=.46	.01 (-.09-.11) p=.96	-.09 (-.20-.01) p=.18	-.08 (-.19-.03) p=.27	-.09 (-.20-.02) p=.20

All analyses controlled for sex, and group comparisons were corrected for multiple testing using a false discovery rate (FDR) procedure. Results in the first column, "sex only", are those presented in the main text, Table 1. *P*-values for significant differences (*p*<.05, FDR corrected) between groups are presented in **bold.** ACEs = adverse childhood experiences, CI = confidence interval, SES = socioeconomic status. Regression coefficients (betas) are standardized coefficients.

eTable 5. Average total brain volume and antisocial group comparisons

	**Mean (SD)**			Comparisons between trajectory groups [β (95% CI), p-value]		
	**Life-course-persistent (N=80)**	**Adolescence-limited (N=151)**	**Low-antisocial (N=441)**	**Life-course-persistent vs. low**	**Adolescence-limited vs. low**	**Life-course-persistent vs. adolescence-limited**
Total Brain Volume	1066193 (109768)	1089075 (113422)	1095974 (116225)	-0.14 (-0.21 - -0.07) p<0.0001	-0.06 (-0.13 - 0.00) p=0.05	-0.12 (-0.23 - -0.12) p=0.02

All volumes in the left-hand section of the table are reported in cubic millimeters (mm^3). All analyses controlled for sex. Regression coefficients (betas) are standardized coefficients.

***eAppendix 2.* Correlations among global and regional MRI measures**

It is expected that subcortical grey-matter volumes will be intercorrelated with one another, and also that these regional volumes will be correlated with total brain volume. eFigure 2 presents heatmaps of the correlations among regional subcortical grey-matter volumes and total brain volume, stratified by sex (as all primary analyses control for sex). Correlation coefficients show that correlations among subcortical regional volumes were varied, ranging from 0.2-0.7. Moreover, correlations of regional subcortical volumes with total brain volume suggest a degree of independence of these regional findings from an overall global effect of total brain volume. Lastly, regional subcortical volume correlations with surface-based cortical measures similarly suggest that, although the general pattern of subcortical grey-matter findings is in line with patterns observed in our previous study of cortical thickness and surface area (8), there is a degree of independence of the subcortical analyses presented here.

eFigure 2. Correlations among regional subcortical grey-matter volumes and total brain volume, global surface area and average cortical thickness

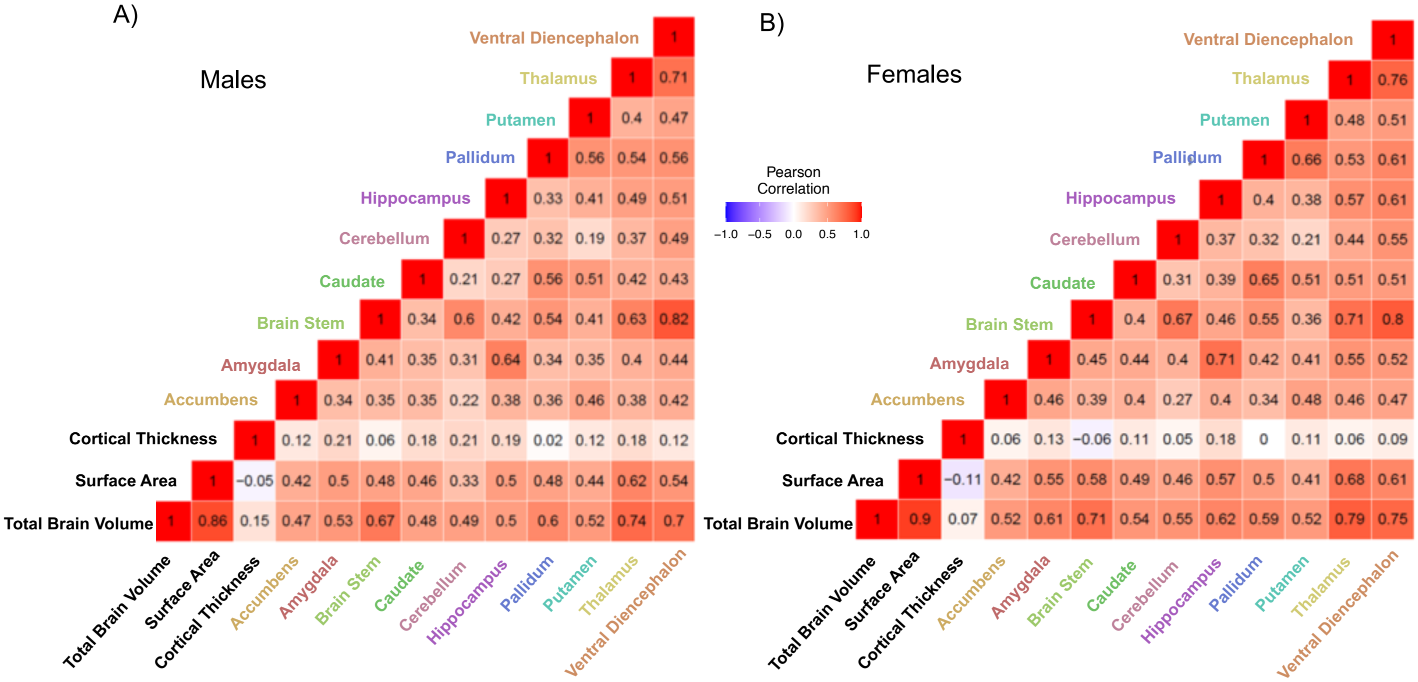

A) Correlation matrix for men, B) correlation matrix for women. Pearson's correlation coefficients are presented for each test. Surface area corresponds to total surface area, cortical thickness corresponds to average cortical thickness.

References

Carlisi, C. O., Moffitt, T. E., Knodt, A. R., Harrington, H., Ireland, D., Melzer, T. R., Poulton, R., Ramrakha, S., Caspi, A., Hariri, A. R., & Viding, E. (2020). Associations between life-course-persistent antisocial behaviour and brain structure in a population-representative longitudinal birth cohort. *The Lancet Psychiatry*, *7*(3), 245–253. https://doi.org/10.1016/S2215-0366(20)30002-X
